# Serum Sphingolipids Reflect the Severity of Chronic HBV Infection and Predict the Mortality of HBV-Acute-on-Chronic Liver Failure

**DOI:** 10.1371/journal.pone.0104988

**Published:** 2014-08-19

**Authors:** Feng Qu, Su-Jun Zheng, Shuang Liu, Cai-Sheng Wu, Zhong-Ping Duan, Jin-Lan Zhang

**Affiliations:** 1 State Key Laboratory of Bioactive Substance and Function of Natural Medicines, Institute of Materia Medica, Chinese Academy of Medical Sciences & Peking Union Medical College, Beijing, China; 2 Artificial Liver Center, Beijing YouAn Hospital, Capital Medical University, Beijing, China; Medical University of South Carolina, United States of America

## Abstract

Patients with HBV-acute-on-chronic liver failure (HBV-ACLF) have high mortality and frequently require liver transplantation; few reliable prognostic markers are available. As a class of functional lipids, sphingolipids are extensively involved in the process of HBV infection. However, their role in chronic HBV infection remains unknown. The aim of this study was to determine the serum sphingolipid profile in a population of patients with chronic HBV infection, paying special attention to exploring novel prognostic markers in HBV-ACLF. High performance liquid chromatography tandem mass spectrometry was used to examine the levels of 41 sphingolipids in 156 serum samples prospectively collected from two independent cohorts. The training and validation cohorts comprised 20 and 28 healthy controls (CTRL), 29 and 23 patients with chronic hepatitis B (CHB), and 30 and 26 patients with HBV-ACLF, respectively. Biometric analysis was used to evaluate the association between sphingolipid levels and disease stages. Multivariate analysis revealed difference of sphingolipid profiles between CHB and HBV-ACLF was more drastic than that between CTRL and CHB, which indicated that serum sphingolipid levels were more likely to associate with the progression HBV-ACLF rather than CHB. Furthermore, a 3-month mortality evaluation of HBV-ACLF patients showed that dhCer(d18∶0/24∶0) was significantly higher in survivors than in non-survivors (including deceased patients and those undergoing liver transplantation, *p*<0.05), and showed a prognostic performance similar to that of the MELD score. The serum sphingolipid composition varies between CTRL and chronic HBV infection patients. In addition, dhCer(d18∶0/24∶0) may be a useful prognostic indicator for the early prediction of HBV-ACLF.

## Introduction

HBV infection is globally endemic. Among the types of chronic HBV infection, HBV-acute-on-chronic liver failure (HBV-ACLF) is one of the most severe end stages [1], [2]. Characteristic features of HBV-ACLF include rapid disease progression and a high incidence (50–90%) of short and medium term mortality [3]. However, the pathogenesis of HBV-ACLF remains unclear [4], [5].

**Figure 1:**
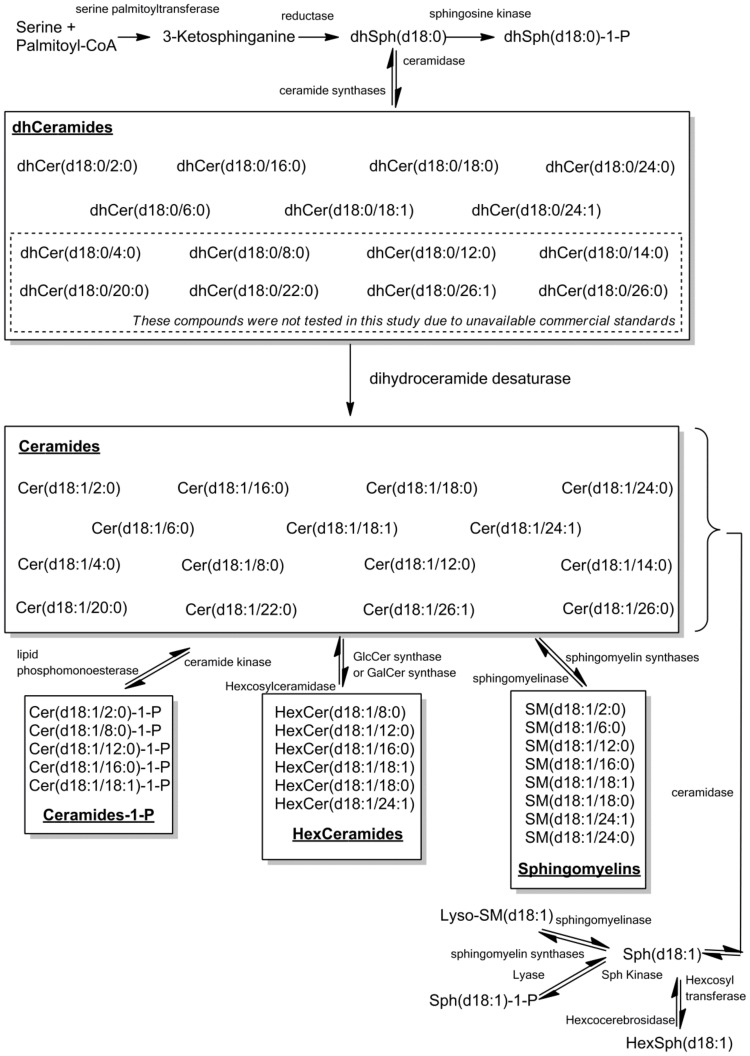
Metabolic pathways of sphingolipids. The sphingolipids examined in this study comprise the core components of sphingolipid metabolism.

All animal viruses rely on constituents of the host cell to provide the energy, macromolecules, and structural organization necessary for survival, and they must cross membranes either by transient local disruption of membrane integrity or by cell lysis [6], [7]. Sphingolipids are highly bioactive compounds that serve as core components of biological structures, such as membranes and lipoproteins; they also regulate cell proliferation, differentiation, interaction, migration, intracellular and extracellular signaling, membrane trafficking, autophagy, and cell death [8]. A number of studies show that sphingolipids are involved in the progression of liver disorders, including viral hepatitis, fibrosis, reperfusion following ischemia (in mice), nonalcoholic steatosis hepatitis, and hepatic cell carcinoma [9]–[15]. Sphingolipid metabolism is highly interconnected, and ceramides and their primary metabolites occupy the central position of the network (**Figure 1**), which is well summarized in recent reviews [16], [17]. Inhibition of hepatitis B virus replication by blocking host sphingolipid biosynthesis has recently been suggested as a therapeutic strategy [15]. Although sphingolipids participate in several aspects of the HBV life cycle, the changes that occur in the serum sphingolipid profile during disease progression, and whether/how these changes play a role in chronic HBV infection, have yet to be defined.

ACLF in patients with chronic HBV infection is increasingly recognized and associated with a poor outcome, with an in-hospital mortality rate of more than 70% if liver transplantation is not possible [5], [18]. Early prognostic prediction is critical for distinguishing patients who require transplantation from those who will survive following intensive medical care alone. Presently, the Child-Pugh score and the Model of End-Stage Liver Disease (MELD) are the most commonly used models to assess the severity of liver disease. However, both of these classification systems were initially designed to evaluate cirrhotic patients. Therefore, the identification of novel prognostic markers remains an important target before a breakthrough appears on HBV-ACLF surveillance and early intervention. Based on the existing results of the study (in the below), we speculated that certain specific sphingolipids are associated with clinical outcomes in ACLF patients.

Because clinical reports on the relationship between sphingolipids and chronic HBV infection are very rare we did not know which one or which subclass of sphingolipids should be our focus. Therefore, with the help of modern metabolomics technologies we first investigated all the sphingolipids of their core network, finding differential metabolites between different stages of chronic HBV infection, and further evaluating their prognostic value in HBV-ACLF. In brief, the aims of the present study were to determine the serum sphingolipid composition in a population of patients with chronic HBV infection, paying special attention to differential sphingolipid metabolites among disease stages, and to further explore novel prognostic markers in HBV-ACLF.

## Methods

### Patient selection

Between July, 2008, and January, 2013, two independent cohorts comprising a total of 108 patients with chronic HBV infection were recruited from Beijing YouAn Hospital (Capital Medical University, Beijing, China). The training cohort comprised 29 patients with chronic hepatitis B (CHB) and 30 patients with HBV-ACLF, whereas the validation cohort comprised 23 patients with CHB and 26 patients with HBV-ACLF. Age- and sex-matched healthy controls (CTRLs) were also enrolled (n = 20 for the training cohort and n = 28 for the validation cohort). CTRLs were community-dwelling individuals who presented for their yearly physical examinations and had no specific complaints or illnesses requiring treatment. All subjects underwent a physical examination, biochemical screening, blood coagulation testing, lipid testing (not available for CTRLs), and liver function testing. 25 of total 52 CHB patients received hepatic puncture biopsy guided by ultrasound. The diseased liver tissues were collected by hepatectomy from the HBV-ACLF patients who underwent the liver transplantation (n = 8). The result of pathologic analysis is shown in **Figure S1**. The baseline characteristics of the all the subjects are summarized in **Table 1**.

**Table 1:** Baseline characteristics of the study subjects.

	Training Cohort (n = 79)				Validation Cohort (n = 77)			
	CTRL	CHB	HBV-ACLF	P value	CTRL	CHB	HBV-ACLF	P value
	(n = 20)	(n = 29)	(n = 30)		(n = 28)	(n = 23)	(n = 26)	
Age: mean (SEM)	37 (3)	37 (2)	39 (2)	0.783	40 (3)	38 (4)	45 (4)	0.435
Gender: M; F (%)	80; 20	69; 31	87; 13	0.200	68; 32	83; 17	85; 15	0.200
ALT (U/L): median (range)	16 (4–31)	75 (13–1988)	83 (37–2400)	0.083	19(3–30)	166(13–782)	683(39–3374)	0.000
AST (U/L): median (range)	20 (13–27)	47 (16–973)	103 (37–1235)	0.023	21(12–27)	108(16–767)	508(57–2599)	0.000
TB (µmol/L): mean (SEM)	13 (0.9)	21 (3)	464 (39)	0.000	11 (1)	24 (5)	390(38)	0.000
AB (g/L): median (range)	45 (41–47)	39 (34-52)	32 (26–41)	0.000	46.2(42–51)	39.7(33–47)	32.0(24–39)	0.000
INR: median (range)	-	0.98(0.88–2.1)	2.1(0.90–5.1)	0.470	-	1.0 (0.93–1.43)	2.2(1.2–3.2)	0.000
HBV DNA (10^3^ cps/mL): median (range)	-	3E4 (1E2–8E8)	5E3 (2E1–4E9)	0.462	-	3E4(5E-1-1E5)	2E4(5E-1-3E5)	0.704
Creatinine (µmol/L): median (range)	-	64 (43–93)	68 (47–590)	0.066	-	74(58–100)	76(40–149)	0.119
PT (s): median (range)	-	12 (10–164)	25 (2–75)	0.014	-	12 (11–16)	26 (2–40)	0.000
PTA (%): mean (SEM)	-	94 (5)	31 (3)	0.000	-	98(4)	34(2)	0.000
WBC (/mm^3^): median (range)	-	5.2 (3–7)	9 (3–113)	0.055	-	5.4(3–9)	8.7 (3–20)	0.002
PLT (/L): median (range)	-	166 (94–297)	91 (35–1107)	0.908	-	154(57–236)	105.4(25–283)	0.000
CHOL (mmol/L): mean (range)	-	4.2 (3–6)	2.3 (0.8–5)	0.000	-	4.2 (3–5)	2.1(1–3)	0.000
TG (mmol/L): mean (range)	-	0.93 (0.33–1.5)	0.69 (0.22–2.9)	0.887	-	0.98 (0.58–1.1)	0.85 (0.3–1.8)	0.715
HDL (mmol/L): mean (range)	-	1.0 (0.88–2.0)	0.30 (0.05–1.2)	0.000	-	1.3 (1.2–2.1)	0.43 (0.06–1.6)	0.000
LDL (mmol/L): mean (range)	-	2.2 (1.1–2.6)	0.95 (0.19–2.7)	0.001	-	2.1 (1.9–2.2)	0.95 (0.32–1.8)	0.000
ApoA1 (g/L): mean (range)	-	96 (89–149)	24 (1.7–71)	0.000	-	109 (76–163)	21 (5.0–46)	0.000
ApoB (g/L): mean (range)	-	65 (50–89)	52 (18–133)	0.351	-	53 (52–56)	53.4 (16.3–84.9)	0.115
A1/B: mean (range)	-	1.7 (1.0–2.3)	0.73 (0.02–1.8)	0.000	-	2 (1.5–3)	0.41 (0.18–2.82)	0.000
Antiviral treatment: (%)		None (76)	None (60)	0.116		None (74)	None (69)	0.151
(before admission)		Enticavir (14)	Enticavir (27)	-		Enticavir (13)	Enticavir (8)	-
		Adefovir dipivoxil (3)	Adefovir dipivoxil (3)	-		Adefovir dipivoxil (9)	Adefovir dipivoxil (8)	-
		Lamivudine (3)	Lamivudine (7)	-		Lamivudine (0)	Lamivudine (4)	-
		Interferon (3)	Interferon (3)	-		Interferon (4)	Interferon (4)	-
		Telbivudine (0)	Telbivudine (0)	-		Telbivudine (0)	Telbivudine (4)	-
MELD: mean (range)	-	-	24(8–55)	-	-	-	24(15–36)	-
Encephalopathy grade: Grade 0; 1; 2; 3; 4 (%)	-	-	53; 30; 10; 7; 0	-	-	-	29; 32; 13; 13; 13	-
Spontaneous bacterial peritonitis: (%)	-	-	65	-	-	-	73	-
Precipitating factors: (%)			HBV reactivation (67)	-			HBV reactivation (70)	-
			Drinking (10)	-			Drinking (5)	-
			Unknown (23)	-			Unknown (25)	-
The basic of ACLF: (%)			Cirrhosis (47)	-			Cirrhosis (65)	-
			CHB (53)	-			CHB (35)	-

Abbreviations: A1/B: apolipoprotein A1 divided by apolipoprotein B; AB, albumin; HBV-ACLF, HBV-acute-on-chronic liver failure; ALT, alanine aminotransferase; AST, aspartate aminotransferase; ApoA1, apolipoprotein A1; ApoB, apolipoprotein B; CHB, chronic hepatitis B; CHOL, cholesterol; HDL, high density lipoprotein; INR, International Normalized Ratio; LC, liver cirrhosis; LDL, low density lipoprotein; MELD, Model for End-Stage Liver Disease; PLT, blood platelet; PT, prothrombin time; PTA, prothrombin activity; TB, total bilirubin; TG, triglyceride; WBC, white blood cell.

### Diagnosis and definitions

All patients included in the study were either positive for HBsAg or HBV DNA (by real-time PCR assay) for more than 6 months before enrollment. Patients with other forms of viral hepatitis, hemochromatosis, Wilson's disease, autoimmune hepatitis, primary biliary cirrhosis, sclerosing cholangitis, biliary obstruction, alpha-1 antitrypsin deficiency, or malignancies were excluded. All CTRLs showed normal liver function and were negative for viral hepatitis; none were alcoholic.

The inclusion criteria for the CHB patients were as follows: all showed a persistent elevation of ALT levels, or repeated elevation of ALT levels and/or evidence of inflammation on histological examination of liver biopsy samples [19].

Patients with HBV-ACLF fulfilled the recommendations for the definition of ACLF established by the Chinese Society of Hepatology [20], in which ACLF is described as the acute decompensation of liver function in patients with chronic pre-existing liver diseases (CHB or cirrhosis in this study). ACLF was defined as the presence of severe jaundice (total bilirubin ≥171 µmol/L), coagulopathy (prolonged prothrombin time, prothrombin activity ≤40%). This diagnostic criteria is mostly consistent with that released by Asian Pacific Association for the study of the liver [5], except the total bilirubin ≥85 µmol/L is required by the later one. Precipitating factors of HBV-ACLF include spontaneous HBV reactivation of CHB, drinking and other unknown reasons. All patients with ACLF received standard ACLF-modifying medical treatment during their hospital stay. This included absolute bed rest, antiviral treatment, intravenous infusion of albumin via a drip, and maintenance of proper hydration and electrolyte and acid-base balance. All patients with HBV-ACLF were followed-up for at least 3 months to record the 3-month mortality rates. The patients who died or underwent liver transplantation during the admission period were recorded directly, and patients who were discharged before the end of the follow-up period were monitored via telephone. Survivors were defined as patients with HBV-ACLF who survived for more than 3 months, whereas non-survivors were defined as patients with HBV-ACLF who died within 3 months or received a liver transplant during this time. The 3 month start point was set as the day on which the serum was collected. The baseline characteristics of the two groups are shown in **Table 2**.

**Table 2:** Baseline characteristics of patients with HBV-ACLF.

	Survivors	Non-survivors	P value
	(n = 25)	(n = 31)	
Age: mean (SEM)	39 (2)	35 (3)	0.394
Gender: M; F (%)	85; 15	88; 12	0.157
ALT (U/L): mean (SEM)	453 (120)	422 (118)	0.857
AST (U/L): median (range)	135 (42–2599)	157 (37–1323)	0.317
TB (µmol/L): mean (SEM)	389 (37)	480 (36)	0.088
AB (g/L): mean (SEM)	33 (1)	31 (1)	0.274
INR: mean (SEM)	2.0 (0.09)	2.4 (0.1)	0.012
HBV DNA (10^3^cps/mL): median (range)	1E5 (1E2–3E8)	5E3 (5E2–4E9)	0.452
Creatinine (µmol/L): mean (SEM)	68 (4)	97 (16)	0.124
PT (s): mean (SEM)	24 (1)	28 (2)	0.158
PTA (%): mean (SEM)	38 (3)	28 (2)	0.007
WBC (/mm^3^): median (range)	8.2 (3.5–113)	7.2 (3.0–66)	0.608
PLT (/L): median (range)	103 (29–856)	80 (25–1107)	0.903
CHOL (mmol/L): mean (SEM)	2.4 (0.2)	2.1 (0.1)	0.228
TG (mmol/L): mean (SEM)	0.96 (0.1)	0.91 (0.1)	0.707
HDL (mmol/L): mean (SEM)	0.47 (0.06)	0.39 (0.07)	0.425
LDL (mmol/L): mean (SEM)	1.2 (0.1)	0.82 (0.08)	0.006
ApoA1 (g/L): mean (SEM)	25 (2)	20 (3)	0.178
ApoB (g/L): mean (SEM)	57 (4)	53 (5)	0.509
A1/B: mean (SEM)	0.47 (0.05)	0.48 (0.1)	0.896
MELD: mean (SEM)	22 (1)	27 (1)	0.004
Encephalopathy grade: Grade 0; 1; 2; 3; 4 (%)	55; 33; 11; 4; 0	32; 29; 12; 15; 12	0.273
Precipitating factors: (%)	HBV reactivation (59)	HBV reactivation (68)	0.159
	Drinking (7)	Drinking (6)	
	Unknown (33)	Unknown (26)	
The basic of ACLF: (%)	Cirrhosis (48)	Cirrhosis (63)	0.261
	CHB (52)	CHB (37)	
Antiviral treatment: (%)	None (56)	None (71)	0.213
(before admission)	Enticavir (15)	Enticavir (20)	
	Adefovir dipivoxil (7)	Adefovir dipivoxil (3)	
	Lamivudine (11)	Lamivudine (6)	
	Interferon (7)	Interferon (0)	
	Telbivudine (4)	Telbivudine (0)	
Antiviral treatment: (%)	Enticavir (68)	Enticavir (77)	0.238
(after admission)	Lamivudine (24)	Lamivudine (16)	
	Adefovir dipivoxil & lamivudine (8)	Adefovir dipivoxil & lamivudine (3)	
	Refuse antiviral treatment (0)	Refuse antiviral treatment (3)	
Other special treatments: (after admission)			0.303
Steroid pulse therapy a (%)	32	45	
Plasmapheresis (%)	80	93	
Immunomodulator therapy b (%)	52	48	

a Medicine: Methylprednisolone.

b Medicine: Thymic peptide α1 or Thymopentin.

The study protocol was reviewed and approved by the Institutional Review Board of Beijing YouAn Hospital. Written informed consent was obtained from each participant before initiation of the study. The study was carried out according to the Declaration of Helsinki and the guidelines of the International Conference on Harmonization for Good Clinical Practice.

### Obtention of serum

Fasted blood samples were prospectively collected in 16×100 mm×10 mL BD Vacutainer glass serum tubes (Becton Dickinson, Franklin Lakes, NJ) in the morning of the second day after the subjects were admitted to the hospital. The tubes were incubated at room temperature for 20 min to allow the blood to clot, and then centrifuged at 750×g for 15 min to obtain the serum. Serum samples were stored at −80°C immediately after collection. Baseline characteristics were acquired on the same day by using the same blood. Sphingolipidomic assays were performed at the Institute of Materia Medica, Peking Union Medical College (Beijing, China).

### Determination of sphingolipids

The high performance liquid chromatography coupled to tandem mass spectrometry (HPLC-MS/MS) was performed using an Agilent 6410B Triple Quad mass spectrometer (Agilent Technologies Inc., Santa Clara, CA) comprising a triple quadrupole MS analyzer equipped with an electrospray ionization interface and an Agilent 1200 RRLC system (HPLC-MS/MS). The HPLC-MS/MS methodology used in this study was described in our previous report [21].

### MELD-based scoring systems

Disease severity in patients with HBV-ACLF was evaluated using the MELD score, which uses the patient's serum bilirubin and creatinine concentrations and the INR for prothrombin time to predict survival. MELD scores were calculated using the web site calculator (http://www.mayoclinic.org/gi-rst/mayomodel7.html).

### Statistical analysis

Sphingolipids were quantified by comparing the peak area ratio (the peak area of the analyte divided by that of its corresponding internal standard) using a standard curve. Analysis was performed on SPSS 18.0 (Chicago, IL, USA) unless otherwise specified. Differences between the groups were analyzed by One-way ANOVA with Tukey post-hoc analyses. For the baseline study, all normally distributed data are expressed as the mean (SEM), and were analyzed by one-way ANOVA (for more than two groups) or an independent sample T-test after Levene's test for equality of variances was performed (for two groups). Non-normally distributed data are expressed as the median (range) and were analyzed by the Kruskal-Wallis test (for more than two groups) or the Mann-Whitney U test (for two groups). Normality was checked by the Shapiro-Wilk test. For categorical variables the chi-square or Fisher's exact test were used. Orthogonal partial least squares discriminant analysis (OPLS-DA), a multivariate analysis method was used to visually discriminate between patients at different HBV disease stages and healthy controls using SIMCA 13.0 software (Umetrics, Umeå, Sweden). Sphingolipidomic data were mean-centered and UV-scaled. The modeling process was run in the auto-fit mode, whereas the components were calculated automatically. Principal component analysis was used to visualize overall clustering or outliers. The quality of each OPLS-DA model was examined using the R2Y(cum) and Q2(cum) values, which are used to assess the stability and predictability of the model, respectively[22]. The criteria for the selection of potential biomarkers were as follows: the value of the variable importance in projection was greater than 1; the jack-knife uncertainty bar excluded zero; and the absolute value of Pcorr in the S-plot was >0.58 [23]. The cross-validation parameter, Q^2^, was calculated as in partial least squares discriminant analysis by permutation testing using 100 random permutations to test the validity of the model against over-fitting. Independent t-test was used to determine whether biomarker candidates obtained from OPLS-DA modeling were significantly different between the groups in the validation cohort. To determine the prognostic value of sphingolipid biomarkers, all patients with ACLF in the two cohorts were combined. Sphingolipids showing marked differences between survivors and non-survivors (identified by independent t-test) were selected as potential predictors. The correlation between the sphingolipid potential predictors and the MELD score was evaluated by Spearman's correlation analysis. The receiver operating characteristic (ROC) curve was obtained and the area under the curve (AUC) was calculated to identify the ability of certain sphingolipid potential predictors and the MELD score to predict 3-month mortality in patients with HBV-ACLF. The Delong method was used to test the significance of the differences between the areas under the ROC curves (MedCalc, Ostend, Belgium) [24]. For all the statistical analyses described above, a two-sided *p* value of <0.05 signified statistical significance.

## Results

### Patient Population

**Table 1** depicts the baseline characteristics of the study subjects from two cohorts. The groups were selected to ensure approximately equal sample size either for groups (n = 20∼30) or for cohorts (n = 70∼80). For each cohort, groups were matched in terms of age and gender (**Table 1**). For all the HBV-ACLF patients, the survivor and non-survivor groups were matched in terms of age, gender, ALT, AST, TB and creatinine (**Table 2**). In addition, significant differences of INR, PTA and MELD between these groups are likely to be explained by they are all reported prognosis marker of ACLF [25]–[27]. The significant decreasing low density lipoprotein (LDL) of non-survivor group can be explained by more cirrhosis based HBV-ACLF patients in this group because it is reported that the decreasing of serum LDL in patients with liver disease was related to the increasing severity of the disease [28]. Antiviral treatment (before admission) may influence the serum sphingolipids, while encephalopathy grade, precipitating factors, the basic of ACLF, antiviral treatment (after admission) and other special treatment (steroid pulse therapy, plasmapheresis and immunomodulator therapy) may influence the outcome of HBV-ACLF. The chi-square or Fisher's exact test shows that there are no significant difference of serum sphingolipids between CHB and HBV-ACLF or between survivors and non-survivors. This means the patient groups were matched in term of antiviral treatment (**Table 2**).

### HPLC-MS/MS profiling identified significant differences and potential biomarkers in serum sphingolipid profiles among the three groups

**Figure 2:**
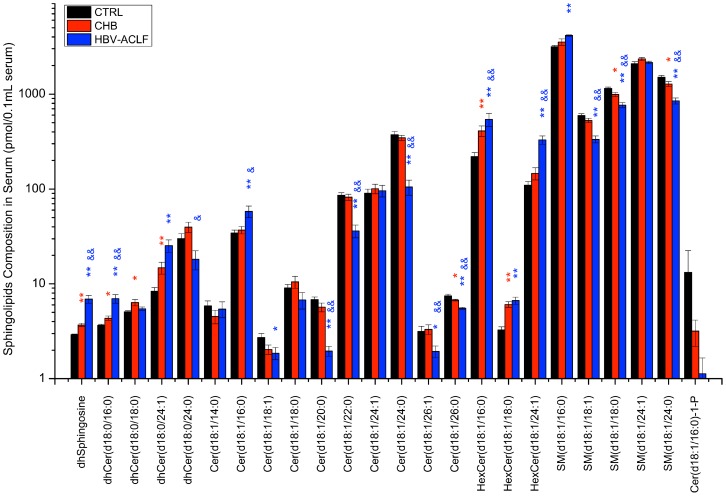
Serum sphingolipid levels in the training cohort (healthy controls, chronic hepatitis patients, and HBV-ACLF patients). Data are expressed as the mean ± SEM. *, significant difference compared with healthy controls (*  = *p*<0.05, **  = *p*<0.01); &, significant difference compared with CHB (&  = *p*<0.05, &&  = *p*<0.01).

We measured the serum sphingolipid profiles in patients with chronic HBV infection by HPLC-MS/MS. In the training cohort, marked differences were observed between groups (**Figure 2**). Detailed data was shown in **Table S1**. In brief, compared with healthy control serum, a total of 10 and 19 sphingolipids were with significant differences in the CHB and HBV-ACLF groups, respectively (*p*<0.05, **Figure 2**). Compared with the CHB group, totally 17 sphingolipids were with significant differences in HBV-ACLF groups (*p*<0.05, **Figure 2**).

**Figure 3:**
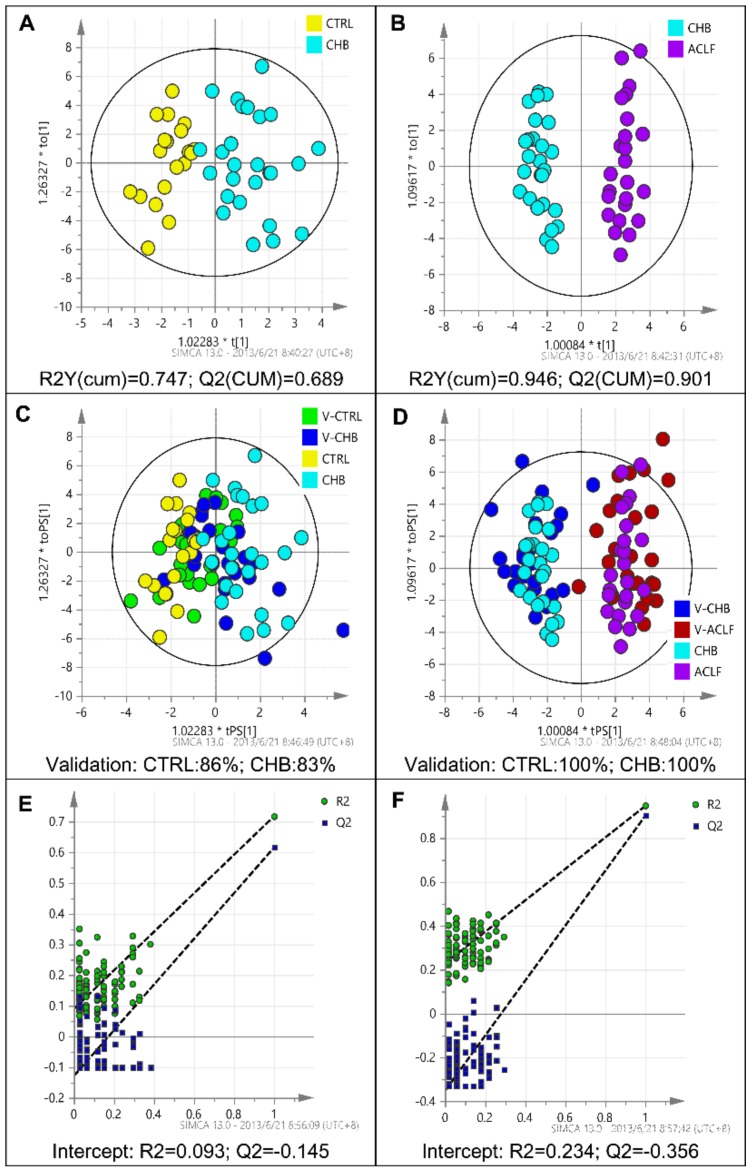
Identification of potential biomarkers through OPLS-DA. (**A**),(**B**). Score plots obtained from the training cohort by OPLS-DA. R2Y(cum) and Q2(cum) values are displayed below each panel; these values have a range of 0 to 1 and represent the stability and predictability of the model, respectively. Dots with different colors representing different groups are well separated based on their sphingolipidome data, indicating serum sphingolipidome varied among groups significantly. (**C**),(**D**). T-predicted scatter plot of the OPLS-DA model using sphingolipidome data obtained from the validation cohort. The result shows that the OPLS-DA models correctly predicted 86% of V-CTRL and 83% of V-CHB patients within the 95% CI (panel C, CTRL vs. CHB), and correctly predicted 100% of V-CHB and 100% of V-CTRL subjects within the 95% CI (panel D, CHB vs. ACLF). (**E**),(**F**). Validation plot of the PLS-DA models obtained using 100 permutation tests to reveal the risk of overfit from the model. The intercept for the blue Q2 line should be below 0.1. Note: V-: corresponding validation group in the validation cohort.

**Figure 4:**
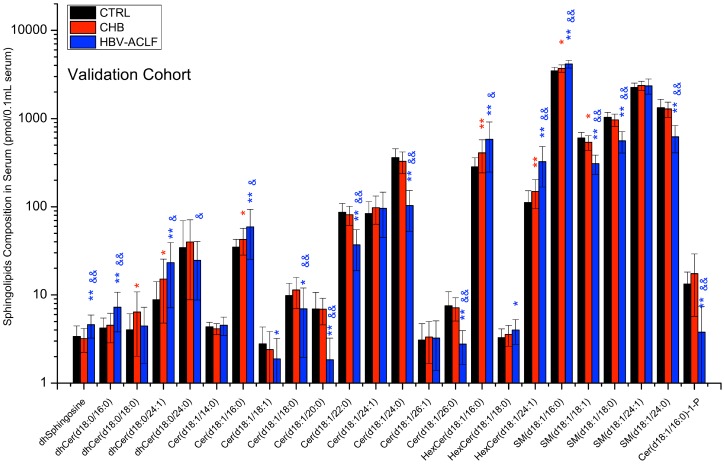
Serum sphingolipid levels in the validation cohort. Data are expressed as the mean ± SEM. *, significant difference compared with healthy control subjects (*  = *p*<0.05, **  = *p*<0.01); &, significant difference compared with CHB (&  = *p*<0.05, &&  = *p*<0.01).

To maximize the separation between sample groups, we performed a supervised multivariate analysis using OPLS-DA. The results revealed distinct clustering between patients at each disease stage in the training cohort (**Figure 3A,B**). According to the criterias used to select potential biomarkers (described in the Methods section), serum obtained from CHB patients could be discriminated from that from healthy controls according to the levels of dhSphingosine and HexCer(d18∶1/18∶0), whereas serum from HBV-ACLF patients could be discriminated from that of CHB patients according to the levels of Cer(d18∶1/20∶0), Cer(d18∶1/22∶0), Cer(d18∶1/24∶0), Cer(d18∶1/26∶0), dhCer(d18∶0/24∶0), dhSphingosine, dhSphingosine-1-P, HexCer(d18∶1/24∶1), and SM(d18∶1/18∶1). After internal cross-validation, all OPLS-DA models showed excellent stability [R2Y(cum)>0.7] and good predictability [Q2(cum)>0.6] **(** **Figure 3A,B**). Validation using 100 random permutation tests generated intercepts of R^2^ = 0.093 and Q^2^ = −0.145 for CTRL versus CHB, and R^2^ = 0.234 and Q^2^ = −0.356 for CHB versu*s* HBV-ACLF (**Figure 3D,E**). The potential biomarkers were then selected for verifying in the validation cohort. A bar-plot of the sphingolipid composition in the validation cohort is shown in **Figure 4** (Detailed data was shown in **Table S2**). The results of independent t-tests (*p*<0.05) between groups excluded all the potential biomarkers identified between the CTRL and CHB groups, whereas all nine potential biomarkers identified between the CHB and HBV-ACLF groups were confirmed; thus, these were regarded as potential, reliable diagnostic biomarkers. Furthermore, sphingolipidomic data obtained from the validation cohort was plotted on the OPLS-DA score plot obtained for the training cohort. As shown in **Figure 3C,D**, the OPLS-DA model correctly predicted 86% of CTRL and 83% of CHB patients within the 95% confidence interval (CI), and correctly predicted 100% of CHB and 100% of CTRL subjects within the 95% CI. This independent external validation confirms the feasibility of LC-MS/MS-based serum sphingolipidomics as a potential diagnostic tool for HBV-ACLF. The results above suggested that sphingolipidome profiles can be used to indicate the progression of liver disease (HBV-ACLF) rather than the development of CHB; this is because nine validated biomarkers could discriminate between CHB and HBV-ACLF patients whereas none could discriminate between CTRL subjects and CHB patients. Thus, we speculated that specific sphingolipids may have some prognostic performance in HBV-ACLF patients, e.g. predicting 3-month mortality, which is a considerable challenge in clinical practice.

### dhCer(d18∶0/24∶0) as a predictor of 3-month mortality in ACLF patients

**Figure 5:**
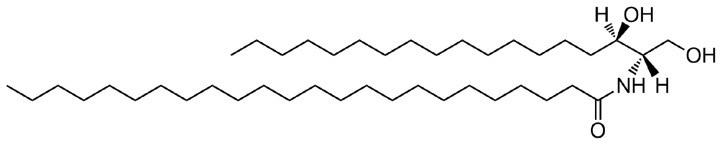
Chemical structure of dhCer(d18∶0/24∶0).

**Figure 6:**
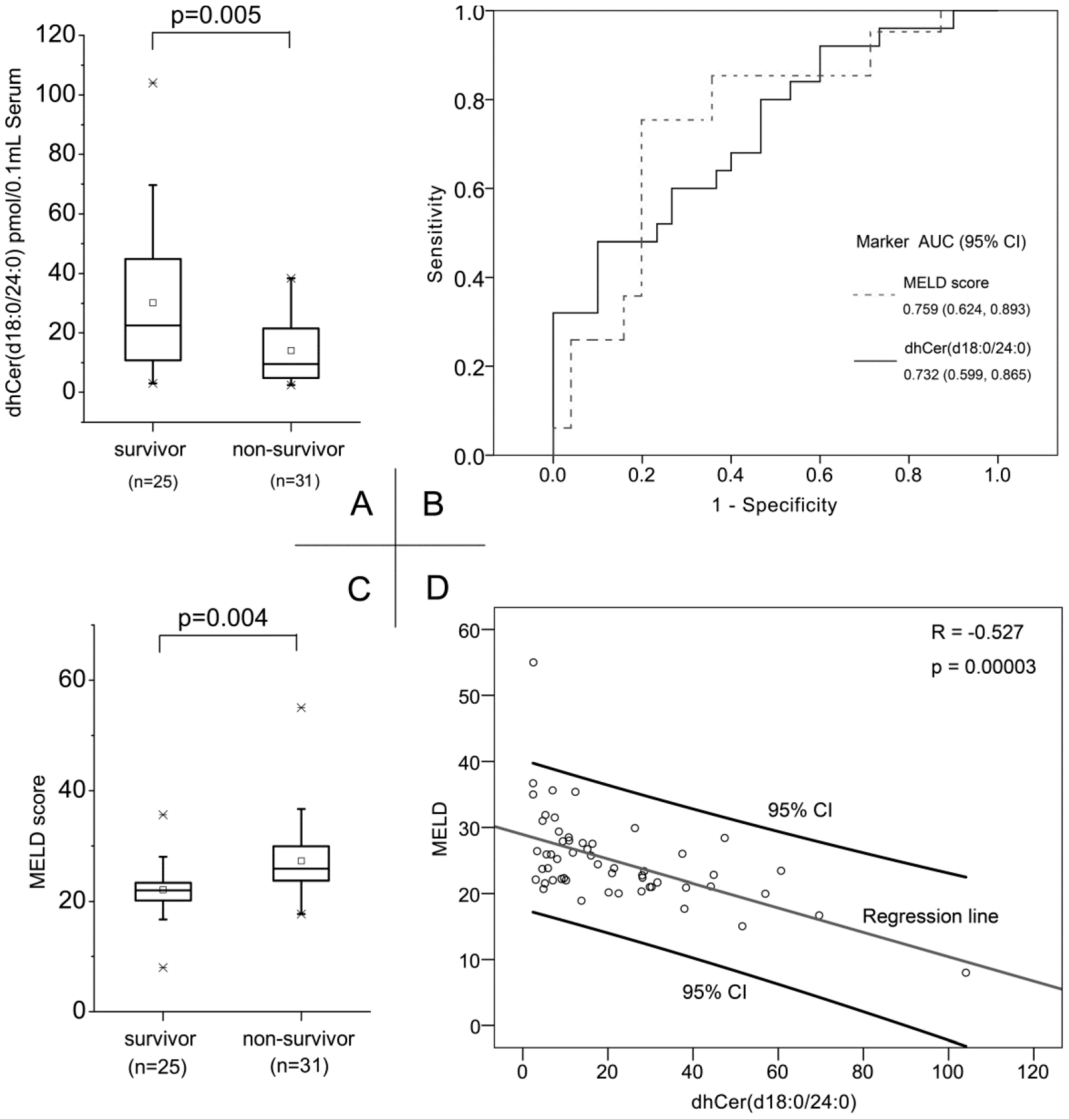
Prognostic performance of dhCer(d18∶0/24∶0) in HBV-ACLF patients in both cohorts. (**A**). The serum levels of dhCer(d18∶0/24∶0) were significantly lower in the non-survivor group compared with those in the survivor group (p = 0.005). Box refers to the 25th and 75th percentile values, with a line indicating median levels, whereas the interquartile range extends outside the box. Points outside the interquartile range are outliers. (**B**). ROC curves of dhCer(d18∶0/24∶0) and MELD score with the 95% CIs, for survivors versus non-survivors in HBV-ACLF patients. (**C**). The MELD scores of non-survivors with HBV-ACLF were significantly higher than those of survivors. (**D**). Serum levels of dhCer(d18∶0/24∶0) in all HBV-ACLF patients significantly and negatively correlated with the MELD scores (R = −0.517, *p* = 0.00003).

Indeed, when all HBV-ALCF patients were divided into two groups, survivors and non -survivors, we observed significant differences in the circulating levels of dhCer(d18∶0/24∶0). The chemical structure of dhCer(d18∶0/24∶0) was shown in **figure 5**. An independent t-test showed that dhCer(d18∶1/24∶0) was the only sphingolipid that showed a significant difference in expression level between survivors and non-survivors (30.2±24.7 pmol/0.1 mL serum in survivors and 14.1±11.4 pmol/0.1 mL serum in non-survivors, *p* = 0.005; **Figure 6A**). This is an excellent indication that decreasing dhCer(d18∶0/24∶0) concentrations may serve as an independent predictor of 3-month mortality in patients with HBV-ACLF. Over the past decade, the MELD score was the most widely used method for predicting mortality and determining organ allocation in liver transplantation [29]. A previous study reported that the model for the MELD score was related to the prognosis of patients with HBV-related ACLF [30]. In the present study, we compared the performance of dhCer(d18∶0/24∶0) levels with that of the MELD scores for predicting 3-month mortality. First, the MELD scores for non-survivors were significantly higher than those of survivors (22.1±5.1 in survivors *vs*. 27.2±7.0 in non-survivors, *p* = 0.004; **Figure 6C**). As shown in **Figure 6B**, ROC analysis revealed that the AUC for dhCer(d18∶0/24∶0) was 0.759 (95% CI: 0.624-0.893), whereas that for the MELD scores was 0.732 (95% CI: 0.599-0.865). Thus, there was no significant statistical difference between the prognostic performance of dhCer(d18∶0/24∶0) levels and that of the MELD scores (*p*>0.05, by Delong method) [24]. Furthermore, Spearman's correlation analysis showed that, among 55 HBV-ACLF patients, dhCer(d18∶0/24∶0) levels significantly correlated with the MELD scores (R = -0.517, *p* = 0.00003; **Figure 6D**). Thus, we conclude that dhCer(d18∶0/24∶0) levels are associated with the severity of liver disease, and exhibit a prognostic performance similar to that of the MELD score. We further conducted logistic regression and model fitting to investigate whether adding dhCER(d18∶0/24∶0) to a prognostic model including MELD improve discrimination. The answer is no. Here is the reason. According to above result, dhCer(d18∶0/24∶0) correlated significantly with MELD (R = -0.527, p = 0.00003). Therefore, both of them represents the severity of liver failure and would not generated more discrimination power to combine them.

## Discussion

Sphingolipids are extensively involved HBV infection pathways and have a significant influence on the life of hepatocytes [15], [31]. This study was designed to examine changes in the serum sphingolipidome of patients with chronic HBV infection, and is the first to demonstrate the utility of serum sphingolipidomic profiling to identify novel prognostic biomarkers of 3-month mortality in patients with HBV-ACLF.

Because perturbations in the levels of certain compounds may initiate a cascade of changes in the levels of multiple lipids, which is called the “ripple effect”, metabolic homeostasis of sphingolipids is key for maintaining the physical health of an organism [16]. To our knowledge, this is the first study to perform serum sphingolipidomic profiling in patients with chronic HBV infection. In the training cohort, multivariate analysis identified potential biomarkers that could discriminate CHB patients from CTRL subjects and HBV-ACLF patients from CHB patients. In the validation cohort, however, all of the potential biomarkers that discriminated CHB patients from HBV-ACLF patients were confirmed, whereas those that potentially differentiated CTRL subjects from CHB patients were screened out. These results suggested that the ripple effect is more significant in patients showing disease progression.

These results allowed us to speculate on the role played by sphingolipids in disease progression. Because the liver plays an essential role in the metabolism of sphingolipids, it is not surprising that liver diseases are associated with major changes in serum sphingolipid concentrations [32]. Progression of HBV-ACLF ultimately leads to increased hepatocyte apoptosis and/or necrosis, which is a hallmark of liver failure. Cellular debris released by necrotic or apoptotic hepatocytes into the circulation may also cause substantial changes in serum sphingolipid composition [33]. Thus, the use of cell death-related sphingolipids to indicate HBV-ACLF status might represent a novel prognostic marker that can be used to better identify patients that require a liver transplant. On the other hand, sphingolipids are extensively involved in the function of the immune system [34]. Increasing evidence suggests that non-HBV-specific inflammation of the liver is likely responsible for the hepatic pathology observed in patients with CHB [35].

Perhaps the most interesting finding from the present study is that decreasing dhCer(d18∶0/24∶0) concentrations can serve as an independent predictor of 3-month mortality in patients with HBV-ACLF. The mechanism underlying the association between serum dhCer(d18∶0/24∶0) concentrations and death in ACLF patients is unclear. However, inflammation in an HBV-infected liver is mediated by cytokines, which are regulated by sphingolipids [36], [37]. The specific cause of this association requires further study. On the other hand, liver failure of other etiologies such as alcohol, acetaminophen, drug-induced hepatitis and autoimmune hepatitis and shock etc. may also have similar associations with serum sphingolipidome. However, it is just a speculation and not our focus. In order to eliminate such uncertainty, we only included HBV induced ACLF patients. Liver failure of other etiologies will be our future focus.

From the perspective of a global metabolomics strategy, metabolites screened with highly differential performance are always identified in a mass to charge ratio form, which could represent whole compounds or fragment ions [38]. Thus, it is necessary to confirm the identity of these metabolites using multi-stage MS or nuclear magnetic resonance. Such confirmation procedures are time-consuming and require significant effort. Here, confirmation was performed in parallel with HPLC-MS/MS quantification, and required no further effort. Sphingolipids were confirmed according to their retention time and fragment pattern that was comparable with that of the reference compounds. Therefore, these results are reliable, and may lead to further metabolic pathway research, or even to the development of simple kits that can be used to evaluate chronic HBV infection.

OPLS-DA is a multivariate classification technique that is used for predicting groupings for observations and for characterizing the groups [39]. The traditional view of disease relationships and subsequent classification is based largely on anatomy and symptoms [40]. OPLS-DA modeling results showed that each stage of chronic HBV infection might have its own sphingolipid profile, or fingerprint. These data-driven efforts to establish disease relationships can offer new opportunities to identify molecular or clinical indices of commonalities or distinctions within each stage of chronic HBV infection. It should be noted that the potential biomarkers identified herein may not be practical for the diagnosis of chronic HBV infection because an easier method already exists (i.e., HBsAg, ALT). Here, we used OPLS-DA to search for potential biomarkers by identifying lipids whose expression levels may correlate with the severity or progression of HBV infection by making full use of the features of OPLS-DA. Nevertheless, it should also be noted that the discovery of a potential biomarker is only the start of the lengthy process of validating them for clinical application or diagnostic purposes.

This study has some limitations. First, it was a single center study and the sample size was relatively small. The findings need to be confirmed in larger multi-center studies before clinical application. Second, due to some economic reasons, details of HBV factors were not tested on all the enrolled patients, for example, genotype, precore mutation and core promoter mutation that were reported as the factors associated with fulminant hepatitis B and may be related to HBV-ACLF [41]. Neither the patients' health insurance nor our grant covered this part. Their relationship with sphingolipids was not revealed in this study. Third, the mechanisms underlying the correlations observed herein remain unclear. Although there were significant differences in the concentrations of many sphingolipids at different stages of chronic HBV infection, the paucity of published clinical reports makes it difficult to connect these results to specific pathological effects and explain their role in HBV development. Given the complexity of sphingolipid metabolism, further *in vitro* and *in vivo* studies are needed.

In conclusion, profiling the sphingolipidome in human serum showed that sphingolipid levels are tightly associated with disease severity in chronic HBV infection. Meanwhile, the data identified reduced serum dhCer(d18∶0/24∶0) concentration as an indicator of poor prognosis in ACLF patients. Assessment of dhCer(d18∶0/24∶0) concentration may have prognostic utility as an early predictor of disease progression, and could contribute to the development of better treatment strategies, such as liver transplantation for patients with low dhCer(d18∶0/24∶0) concentrations in order to reduce their pain and improve the cure rate and intensive medical care alone for patients with high dhCer(d18∶0/24∶0) concentrations in order to reduce the waste of the precious donated livers. However, such decision needs comprehensive evaluating the patient's situation. The procedure can be further optimized to measure dhCer(d18∶0/24∶0) specifically and more rapidly, and can be included in the routine clinical evaluation of patients with HBV-ACLF. This would also allow clinicians to evaluate the prognosis of HBV-ACLF patients in most clinical settings, and potentially improve the reliability of the MELD score rankings.

## Supporting Information

Figure S1 **Histology of liver tissue samples in representative patients.** (**A**) **CHB, mild.** Moderate interface hepatitis with enlarged portal tract. Spot necrosis is in the lobule (left, HE×100). Portal tract shows mild fibrosis with short thin septa (right, Masson trichrome ×100). (**B**) HBV-**ACLF based on CHB.** Massive necrosis of parenchyma (left, HE×100) without cirrhotic nodule (right, Masson trichrome ×100). (**C**) **HBV-ACLF based on cirrhosis.** Massive necrosis of parenchyma with cirrhotic nodule remaining (left, HE×100). Cirrhotic nodule is surrounded by fibrous tissue (Arrow) (right, Masson trichrome ×100).
(DOCX)

Table S1 **Serum sphingolipidome of training cohort.**
(DOCX)

Table S2 **Serum sphingolipidome of validation cohort.**
(DOCX)
