# Supplementary material for: Serum Sphingolipids Reflect the Severity of Chronic HBV Infection and Predict the Mortality of HBV-Acute-on-Chronic Liver Failure
Source: PLoS One. 2014 Aug 19;9(8):e104988. doi: 10.1371/journal.pone.0104988 (PMC4138167; doi:10.1371/journal.pone.0104988)

**Figure S1. Histology of liver tissue samples in representative patients.** **(A) CHB, mild.** Moderate interface hepatitis with enlarged portal tract. Spot necrosis is in the lobule (left, HE ×100). Portal tract shows mild fibrosis with short thin septa (right, Masson trichrome ×100). **(B)** HBV-**ACLF based on CHB.** Massive necrosis of parenchyma (left, HE ×100) without cirrhotic nodule (right, Masson trichrome ×100). **(C) HBV-ACLF based on cirrhosis.** Massive necrosis of parenchyma with cirrhotic nodule remaining (left, HE ×100). Cirrhotic nodule is surrounded by fibrous tissue (Arrow) (right, Masson trichrome ×100).


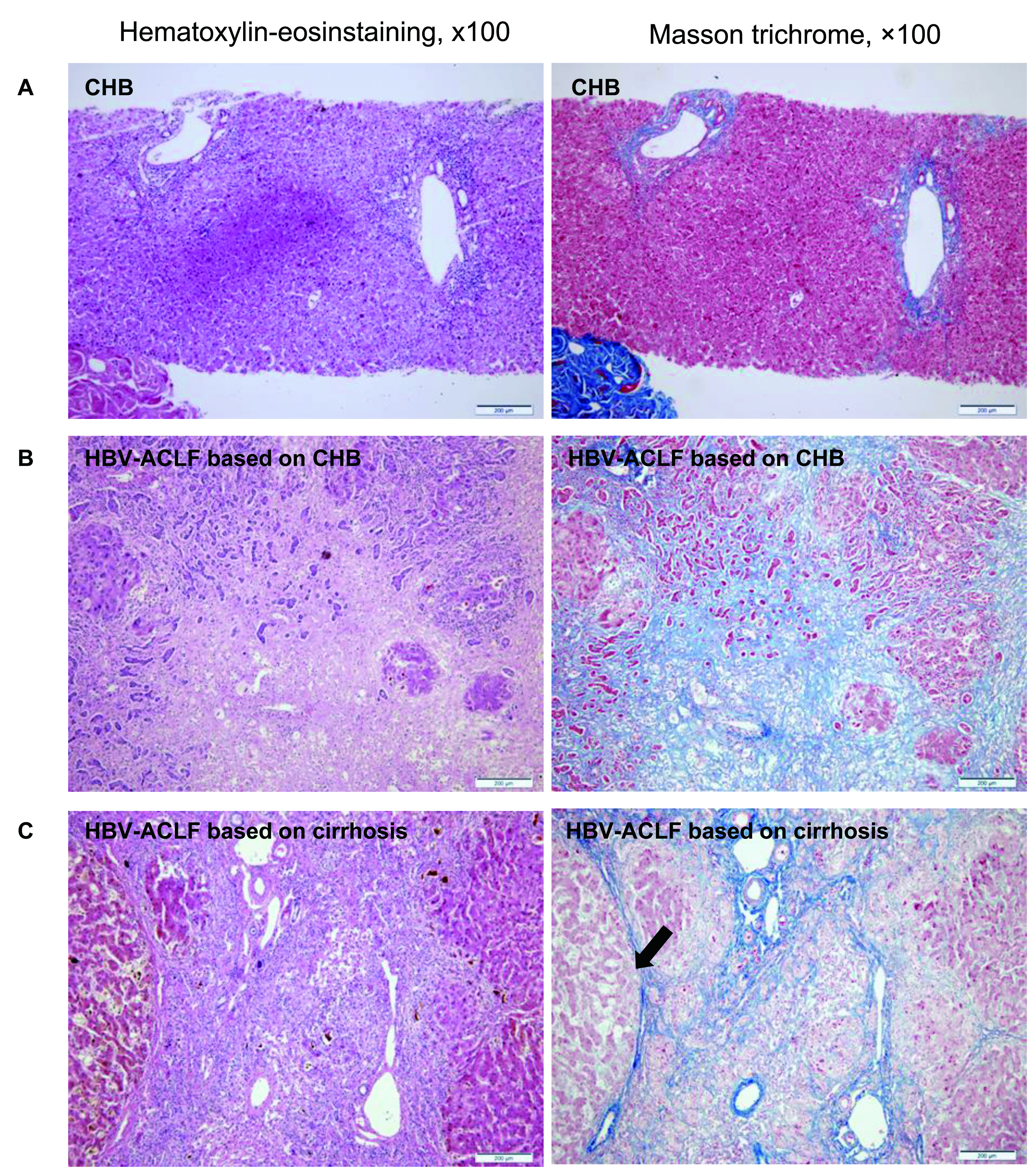

Supplement: Figure S1 — Histology of liver tissue samples in representative patients. (A) CHB, mild. Moderate interface hepatitis with enlarged portal tract. Spot necrosis is in the lobule (left, HE×100). Portal tract shows mild fibrosis with short thin septa (right, Masson trichrome ×100). (B) HBV-ACLF based on CHB. Massive necrosis of parenchyma (left, HE×100) without cirrhotic nodule (right, Masson trichrome ×100). (C) HBV-ACLF based on cirrhosis. Massive necrosis of parenchyma with cirrhotic nodule remaining (left, HE×100). Cirrhotic nodule is surrounded by fibrous tissue (Arrow) (right, Masson trichrome ×100). (DOCX) [file pone.0104988.s001.docx]
