# Supplementary material for: Serum Sphingolipids Reflect the Severity of Chronic HBV Infection and Predict the Mortality of HBV-Acute-on-Chronic Liver Failure
Source: PLoS One. 2014 Aug 19;9(8):e104988. doi: 10.1371/journal.pone.0104988 (PMC4138167; doi:10.1371/journal.pone.0104988)
Supplement: Table S1 — Serum sphingolipidome of training cohort. (DOCX) [file pone.0104988.s002.docx]

Table S1. Serum sphingolipidome of training cohort.

| **Sphingolipid** |  | **CTRL** | |  | **CHB** | | |  | **HBV-ACLF** | | | |
| --- | --- | --- | --- | --- | --- | --- | --- | --- | --- | --- | --- | --- |
|  |  | **Mean** | **SEM** |  | **Mean** | **SEM** | **Sig** |  | **Mean** | **SEM** | **Sig** | **Sig** |
| dhSphingosine |  | 2.92 | 0.04 |  | 3.69 | 0.15 | ** |  | 6.91 | 0.67 | ** | && |
| dhCer(d18:0/16:0) |  | 3.65 | 0.12 |  | 4.34 | 0.23 | * |  | 6.98 | 0.72 | ** | && |
| dhCer(d18:0/18:0) |  | 5.08 | 0.14 |  | 6.35 | 0.49 | * |  | 5.47 | 0.23 |  |  |
| dhCer(d18:0/24:1) |  | 8.35 | 0.75 |  | 14.75 | 2.12 | ** |  | 25.26 | 3.89 | ** |  |
| dhCer(d18:0/24:0) |  | 30.10 | 3.74 |  | 39.73 | 5.04 |  |  | 18.17 | 4.18 |  | & |
| Cer(d18:1/14:0) |  | 5.86 | 0.74 |  | 4.54 | 0.74 |  |  | 5.43 | 1.04 |  |  |
| Cer(d18:1/16:0) |  | 34.39 | 2.58 |  | 37.02 | 3.27 |  |  | 58.22 | 8.24 | ** | & |
| Cer(d18:1/18:1) |  | 2.72 | 0.28 |  | 2.04 | 0.23 |  |  | 1.86 | 0.27 | * |  |
| Cer(d18:1/18:0) |  | 9.05 | 0.78 |  | 10.49 | 1.50 |  |  | 6.76 | 1.33 |  |  |
| Cer(d18:1/20:0) |  | 6.82 | 0.44 |  | 5.68 | 0.61 |  |  | 1.96 | 0.24 | ** | && |
| Cer(d18:1/22:0) |  | 85.94 | 5.62 |  | 82.20 | 6.09 |  |  | 36.21 | 5.54 | ** | && |
| Cer(d18:1/24:1) |  | 90.27 | 9.49 |  | 100.74 | 11.71 |  |  | 96.04 | 13.46 |  |  |
| Cer(d18:1/24:0) |  | 372.41 | 30.57 |  | 346.37 | 21.97 |  |  | 105.02 | 19.29 | ** | && |
| Cer(d18:1/26:1) |  | 3.15 | 0.41 |  | 3.31 | 0.39 |  |  | 1.94 | 0.28 | * | && |
| Cer(d18:1/26:0) |  | 7.47 | 0.31 |  | 6.75 | 0.14 | * |  | 5.52 | 0.14 | ** | && |
| HexCer(d18:1/16:0) |  | 219.80 | 22.45 |  | 410.39 | 51.83 | ** |  | 542.04 | 85.02 | ** | && |
| HexCer(d18:1/18:0) |  | 3.27 | 0.25 |  | 6.06 | 0.43 | ** |  | 6.67 | 0.58 | ** |  |
| HexCer(d18:1/24:1) |  | 109.82 | 10.20 |  | 146.41 | 21.39 |  |  | 329.26 | 33.48 | ** | && |
| SM(d18:1/16:0) |  | 3151.75 | 104.13 |  | 3533.19 | 275.38 |  |  | 4139.64 | 72.03 | ** | & |
| SM(d18:1/18:1) |  | 596.00 | 25.94 |  | 527.45 | 28.21 |  |  | 333.90 | 27.74 | ** | && |
| SM(d18:1/18:0) |  | 1146.11 | 37.23 |  | 991.92 | 49.67 | * |  | 764.76 | 46.39 | ** | && |
| SM(d18:1/24:1) |  | 2094.33 | 97.55 |  | 2345.30 | 91.55 |  |  | 2154.75 | 64.52 |  |  |
| SM(d18:1/24:0) |  | 1501.54 | 70.21 |  | 1277.90 | 76.34 | * |  | 848.54 | 62.80 | ** | && |
| Cer(d18:1/16:0)-1-P |  | 13.20 | 9.17 |  | 3.18 | 0.98 |  |  | 1.12 | 0.53 |  |  |

*p<0.05 and **p<0.01, significant difference compared with CTRL; &p<0.05 and &&p<0.01, significant difference compared with CHB.

Unit of lipid levels: pmol/0.1 mL plasma
