# Supplementary material for: Serum Sphingolipids Reflect the Severity of Chronic HBV Infection and Predict the Mortality of HBV-Acute-on-Chronic Liver Failure
Source: PLoS One. 2014 Aug 19;9(8):e104988. doi: 10.1371/journal.pone.0104988 (PMC4138167; doi:10.1371/journal.pone.0104988)
Supplement: Table S2 — Serum sphingolipidome of validation cohort. (DOCX) [file pone.0104988.s003.docx]

Table S2. Serum sphingolipidome of validation cohort.

| **Sphingolipid** |  | **CTRL** | |  | **CHB** | | |  | **HBV-ACLF** | | | |
| --- | --- | --- | --- | --- | --- | --- | --- | --- | --- | --- | --- | --- |
|  |  | **Mean** | **SEM** |  | **Mean** | **SEM** | **Sig** |  | **Mean** | **SEM** | **Sig** | **Sig** |
| dhSphingosine |  | 3.39 | 1.06 |  | 3.20 | 0.97 |  |  | 4.59 | 1.34 | ** | ## |
| dhCer(d18:0/16:0) |  | 4.22 | 1.25 |  | 4.53 | 1.67 |  |  | 7.25 | 3.45 | ** | ## |
| dhCer(d18:0/18:0) |  | 4.02 | 2.09 |  | 6.41 | 4.39 | * |  | 4.45 | 2.79 |  |  |
| dhCer(d18:0/24:1) |  | 8.83 | 5.26 |  | 15.11 | 10.30 | ** |  | 23.19 | 16.04 | ** | # |
| dhCer(d18:0/24:0) |  | 34.27 | 35.37 |  | 39.96 | 31.18 |  |  | 24.63 | 15.88 |  | # |
| Cer(d18:1/14:0) |  | 4.36 | 0.55 |  | 4.13 | 0.59 |  |  | 4.54 | 1.06 |  |  |
| Cer(d18:1/16:0) |  | 34.79 | 8.13 |  | 42.68 | 14.43 | * |  | 59.33 | 34.08 | ** | # |
| Cer(d18:1/18:1) |  | 2.79 | 1.54 |  | 2.41 | 1.43 |  |  | 1.88 | 1.33 | * |  |
| Cer(d18:1/18:0) |  | 9.82 | 3.70 |  | 11.38 | 4.38 |  |  | 6.96 | 4.99 | * | ## |
| Cer(d18:1/20:0) |  | 6.93 | 3.75 |  | 6.88 | 2.29 |  |  | 1.84 | 1.41 | ** | ## |
| Cer(d18:1/22:0) |  | 86.80 | 22.25 |  | 81.24 | 19.95 |  |  | 36.83 | 18.10 | ** | ## |
| Cer(d18:1/24:1) |  | 83.83 | 30.39 |  | 97.74 | 34.32 |  |  | 95.70 | 50.95 |  |  |
| Cer(d18:1/24:0) |  | 361.01 | 91.72 |  | 326.95 | 89.90 |  |  | 103.00 | 50.11 | ** | ## |
| Cer(d18:1/26:1) |  | 3.09 | 1.63 |  | 3.33 | 1.67 |  |  | 3.24 | 1.86 |  |  |
| Cer(d18:1/26:0) |  | 7.52 | 3.35 |  | 7.16 | 2.11 |  |  | 2.78 | 1.16 | ** | ## |
| HexCer(d18:1/16:0) |  | 283.45 | 74.97 |  | 407.82 | 166.48 | ** |  | 582.14 | 334.50 | ** | # |
| HexCer(d18:1/18:0) |  | 3.29 | 0.82 |  | 3.56 | 0.94 |  |  | 4.01 | 1.26 | * |  |
| HexCer(d18:1/24:1) |  | 111.42 | 40.42 |  | 149.13 | 54.06 | ** |  | 324.09 | 156.91 | ** | ## |
| SM(d18:1/16:0) |  | 3467.63 | 344.32 |  | 3701.73 | 360.20 | * |  | 4150.24 | 418.82 | ** | ## |
| SM(d18:1/18:1) |  | 601.02 | 92.63 |  | 536.35 | 101.42 | * |  | 308.15 | 75.68 | ** | ## |
| SM(d18:1/18:0) |  | 1036.68 | 143.24 |  | 968.33 | 158.73 |  |  | 556.50 | 150.12 | ** | ## |
| SM(d18:1/24:1) |  | 2250.23 | 276.38 |  | 2370.56 | 280.40 |  |  | 2354.24 | 462.32 |  |  |
| SM(d18:1/24:0) |  | 1331.75 | 326.36 |  | 1284.06 | 253.09 |  |  | 619.41 | 211.09 | ** | ## |
| Cer(d18:1/16:0)-1-P |  | 13.29 | 4.88 |  | 17.47 | 11.74 |  |  | 3.80 | 3.67 | ** | ## |

*p<0.05 and **p<0.01, significant difference compared with CTRL; &p<0.05 and &&p<0.01, significant difference compared with CHB.

Unit of lipid levels: pmol/0.1 mL plasma
